# Moving from qPCR to Chip Digital PCR Assays for Tracking of some *Fusarium* species causing *Fusarium* Head Blight in Cereals

**DOI:** 10.3390/microorganisms8091307

**Published:** 2020-08-27

**Authors:** Caterina Morcia, Giorgio Tumino, Giulia Gasparo, Caterina Ceresoli, Chiara Fattorini, Roberta Ghizzoni, Paola Carnevali, Valeria Terzi

**Affiliations:** 1 Council for Agricultural Research and Economics, Research Centre for Genomics and Bioinformatics, I-29017 Fiorenzuola d’Arda PC, Italy; caterina.morcia@crea.gov.it (C.M.); giorgiotumino@hotmail.it (G.T.); giulia.gasparo@gmail.com (G.G.); caterinaceresoli@gmail.com (C.C.); chiarafattorini@alice.it (C.F.); roberta.ghizzoni@crea.gov.it (R.G.); 2 Barilla S.p.A., I-43122 Parma PR, Italy; paola.carnevali@barilla.com

**Keywords:** molecular diagnostics, *Fusarium*, chip digital PCR, qPCR

## Abstract

*Fusarium* Head Blight (FHB) is one of the major diseases affecting small-grain cereals, worldwide spread and responsible for severe yield and quality losses annually. Diagnostic tools, able to track *Fusarium* species even in the early stages of infection, can contribute to mycotoxins’ risk control. Among DNA-based technologies for *Fusarium* detection, qPCR (single and multiplex assays) is currently the most applied method. However, pathogen diagnostics is now enforced by digital PCR (dPCR), a breakthrough technology that provides ultrasensitive and absolute nucleic acid quantification. In our work, a panel of chip digital PCR assays was developed to quantify *Fusarium graminearum*, *F.culmorum*, *F. sporotrichioides*, *F. poae* and *F. avenaceum*. The primers/probes combinations were evaluated on pure fungal samples with cdPCR technique, in comparison with the qPCR approach. Moreover, the cdPCR assays were applied to quantify *Fusarium* in durum wheat and oat samples, naturally contaminated or spiked with fungal DNA. For a better evaluation of infection level in plants, duplex assays were developed, able to co-amplify both plant and fungal DNA. To the best of our knowledge, this is the first study directed to the application of digital PCR to *Fusarium* diagnosis in plants.

## 1. Introduction

Fusarium Head Blight (FHB) is one of the major diseases affecting small-grain cereals, it is worldwide spread and responsible for severe yield and quality losses annually.

Several fungal species, mainly of the *Fusarium* genus, have been identified as the etiological agents of such a disease. In European cultivation environments, FHB occurs, mainly, because of *Fusarium graminearum* and *Fusarium culmorum* presence, but also *Fusarium poae*, *Fusarium pseudograminearum*, *Fusarium avenaceum*, *Fusarium sporotrichioides* and *Fusarium langsethiae*. Most of the species associated with FHB, in advantageous environmental conditions, invade the ear of the cereals and produce toxic secondary metabolites—mycotoxins—that contaminate the grain. FHB, therefore, compromises not only the yield but also the grain safety and quality due to the accumulation of mycotoxins in infected kernels. Depending on species and chemotypes, *Fusarium* can produce A and B trichothecenes: type A trichothecenes include highly toxic mycotoxins, such as T-2 and HT-2, meanwhile type B trichothecenes include, among others, deoxynivalenol (DON), nivalenol (NIV) and acetyl-NIV. Moreover, fumonisins, zearalenone, beauvericin and enniatin B can accumulate in cereal grains after *Fusarium* infection. All these fungal secondary metabolites can cause a wide range of diseases as well as death in humans and animals [1].

Although contamination from mycotoxins occurs in the field, it has repercussions throughout the whole supply chain, including storage periods and transformation steps in the agri-food industries. Physical, chemical, and biological treatments might be defective for the purpose of total decontamination and/or detoxification, with permanence of active mycotoxins along the production cycle as consequence [2].

The fungal load, an environment conducive to the fungus development and a suitable host are indispensable prerequisites for the success of *Fusarium* infection. Consequently, several strategies can be activated to counter FHB. From an agronomic point of view, the fungal load can be reduced adopting Good Agricultural Practices (GAPs) [3]: e.g., optimizing the seeding density and fertilization levels, introducing crop rotation and soil practices that reduce crop residues [4]. The effectiveness of fungicidal treatments is related to their administration coinciding with the restricted period in which the initial infection of the plant can occur [5]. Starting from the information collected in monitoring actions, prediction models were developed. Such models, by weighing the various environmental and agronomic factors, give predictive indications on the risk of infection in cultivation areas [6]. To these strategies, the genetic one is implemented as well, that aims at obtaining, through different breeding approaches, naturally resistant varieties and, at the same time, maintaining the environmental impact of agriculture as low as possible [7]. The epidemiological characterization of present and emerging fungal species is also a useful tool in mycotoxins’ risk control, together with the possibility of having facile, affordable, and early applicable diagnostic methods.

**Figure 1:**
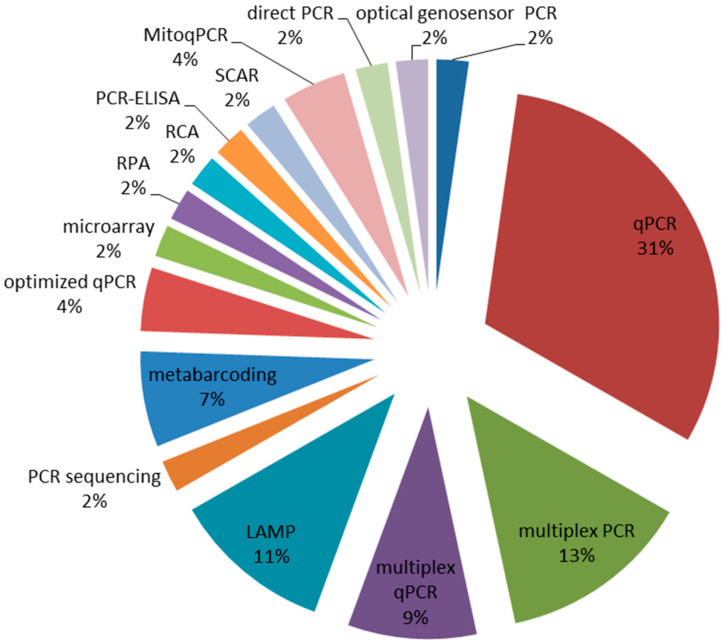
Relative percentages of molecular technologies currently applied to *Fusarium* detection in small-grain cereals. The data have been extracted from a panel of 50 peer-reviewed publications spanning 2009–2019 and specifically focused on the use of DNA tracking for *Fusarium* diagnosis in small-grain cereals [9].

A molecular diagnostic approach has been proposed as an alternative strategy to traditional microbiological techniques for the identification and quantification of *Fusarium* species [8]. A bibliographic search, focusing on DNA-based methods and aiming to conduct *Fusarium* diagnostics in small-grain cereals, was published in 2009–2019 [9]. By applying the appropriate filters, 50 publications have been selected and analyzed to derive information, among others, on the molecular technology used. The obtained results are schematically shown in Figure 1, from which it can be inferred that the qPCR (single and multiplex assays) is the most widespread method, followed by multiplex PCR, LAMP-based protocols and metabarcoding. 

Phytopathogen diagnostics can be now enforced by digital PCR (dPCR), a new PCR application in which the reaction volume is split over a high number of small-volume partitions or droplets [10]. After end-point amplification, each partition can be positive or negative, depending on the presence of a target sequence, therefore giving a binary or digital read-out. Poisson statistics are then used to determine the absolute quantity of target DNA in a sample. It is an absolute quantification strategy, therefore there is no need to have a standard curve reference. Moreover, the end-point measurement enables quantification independently of the reaction efficiency, thereby digital PCR can be used for low-target quantification even in variable contaminated samples [11,12]. Digital PCR is now considered an important tool in plant pathology laboratories [13] both for diagnostics [14] and pathogen biology studies [15]. The aim of our work was to develop a panel of dPCR assays to identify and quantify *Fusarium* species widely spread in cereals crop, starting from qPCR-based assays. The chip digital PCR (cdPCR)-developed assays were evaluated in durum wheat and oat samples naturally contaminated or spiked with *Fusarium* DNA. To the best of our knowledge, dPCR assays have not been evaluated until now for *Fusarium* diagnosis. 

## 2. Materials and Methods 

### 2.1. Fungal Samples

*F.culmorum* (MPVP/70) and *F. avenaceum* (MPVP/66) strains were obtained from Università Cattolica del Sacro Cuore, Piacenza, Italy. *F. graminearum* (ITEM 6477), *F. sporotrichioides* (ITEM 194) and *F. poae* (ITEM 10402) were provided by ISPA, Institute of Sciences of Food Production, CNR-National Research Council, Bari, Italy and belong to the ISPA collection of toxigenic fungi of agro-food interest (www.ispa.cnr.it/Collection).

Strains were stored on potato dextrose agar (PDA, Liofilchem, Teramo, Italy) at 4 °C until use. Fungal DNA was extracted from lyophilized mycelium previously grown on PDA medium, according to the procedure described by Al-Samarrai and Schmid [16]. DNA concentrations were determined using Qubit^®^ fluorimeter (Life Technologies™, Invitrogen, Monza, Italy)

### 2.2. Plant Samples

In the study 19 *Triticum durum* (cultivars Claudio, Simeto, Aureo, Svevo and Creso) and 4 *Avena sativa* (cultivars Buffalo and Tardis) grain samples were used. 

The plants were grown in the experimental fields of Research Centre for Genomics and Bioinformatics, in the 2015 and 2016 seasons, without any fungicide treatment. The 23 wheats and oats were grown in 3-m^2^ plots, in triplicate. At maturity, the plots were harvested, and 20 gr of grains were sampled from each of the three plots and bulked. The 60-gr bulked sample was then milled into a fine powder using an analytical mill (IKA Universal mill M20, IKA-Werke GmbH, Staufen, Germany) and stored at 4 °C until analysis.

Plant genomic DNA was extracted in triplicate from 100 mg samples from the 60-gr bulked milled grains using DNeasy Plant Mini Kit (Qiagen Italia, Milano, Italy) according to the manufacturer’s instructions. DNA concentrations were determined using Qubit^®^ fluorimeter (Life Technologies™, Invitrogen, Monza, Italy). The DNA extracted were analyzed with qPCR to evaluate the presence of *Fusarium*. A subset of these samples was analyzed with cdPCR for *Fusarium* quantification. Moreover, a second subset of plant DNA samples was spiked with fungal DNA. For the preparation of such samples, batches of 20 ng plant DNA were added with 250, 100, 10, and 1 pg of fungal DNA.

### 2.3. Design of Primers and Probes

Table 1 reports the primers and probes sequences. Primer Express 3.0.1 Software (Life Technologies™, Invitrogen, Monza, Italy) was used to design *F. spo*, *F. gram/culm* and Avena dig assays. Multiple Primer Analyzer (Thermo Fisher Scientific, Monza, Italy) was used to verify the absence of self-complementarity and primer dimer formation. 

### 2.4. qPCR

Real-time reactions were prepared in duplicate with 8 μL of QuantStudioTM 3D Digital PCR 2X Master Mix (Applied Biosystems by Life Technologies, Monza, Italy), 900 nM forward and reverse primers, 200nmol of FAM and VIC-MGB probes, 20 ng of DNA template and water to 16 μL. The amplifications were done in a 7300 RealTime PCR System (Applied Biosystems, Thermo Fisher Scientific, Waltham, MA, USA). The best amplification conditions obtained after optimization step for all assays are reported in Table 2. 

For the determination of reaction efficiencies, standard curves were generated by plotting the Ct (Cycle Threshold) values versus the log10 amount of pure DNA of the different *Fusarium* (10-fold dilution series).

### 2.5. Chip Digital PCR

QuantStudioTM 3D Digital PCR System (Applied Biosystems by Life Technologies, Monza, Italy) was used for Chip digital PCR assays. The reaction was done in a final volume of 16 μL obtained by mixing 8 μL of QuantStudioTM 3D Digital PCR 2X Master Mix, 0.72 μL of each primer at 20 μM (final concentration 900 nmol), 0.32 μL of FAM and VIC-MGB probes at 10 μM (final concentration 200 nmol), 2 μL of DNA and nuclease free-water. Nuclease-free water as template was used in the negative control. The reaction mixture of 15 μL was loaded onto the QuantStudioTM 3D Digital PCR chips using QuantStudioTM 3D Digital chip loader, according to manufacturer instructions. Amplifications were performed in ProFlexTM 2Xflat PCR System Thermocycler (Applied Biosystems by Life Technologies, Monza, Italy) under the same conditions used for qPCR amplifications and reported in Table 2. End-point fluorescence data were collected in QuantStudioTM 3D Digital PCR Instrument and files generated were analyzed using cloud-based platform QuantStudioTM 3D AnalysisSuite dPCR software, version 3.1.6. Each sample was analyzed in duplicate.

## 3. Results

### 3.1. Fungal Samples

The fungal DNA stocks were initially quantified with Qubit and the same dilutions of *F. sporotrichioides*, *F. graminearum, F. culmorum*, *F. poae* and *F. avenaceum* DNA were amplified with both qPCR and cdPCR techniques. A dynamic range of 0.5–0.0005 ng of fungal DNA was considered in all the assays. The same primers/probe combinations and the same amplification conditions were applied in both techniques. Table 3 reports the qPCR assays’ efficiencies, as determined with qPCR.

**Figure 2:**
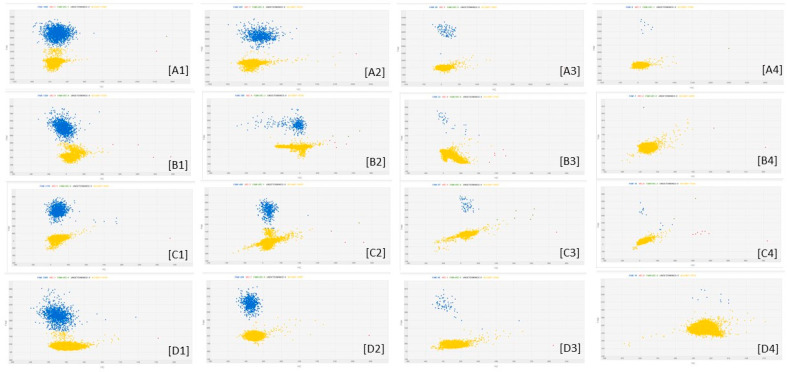
cdPCR amplification plots of *Fusarium* DNA dilutions. The letters indicate the different fungal species: [**A**] = *F. poae*; [**B**] = *F. sporotrichioides*; [**C**] = *F. graminearum*; [**D**] = *F. avenaceum*. The numbers indicate the dilutions factors: 1 means 0.25 ng of fungal DNA as Qubit quantified; 2 means sample 1 diluted 2.5 times; 3 means sample 2 diluted 10 times; 4 means sample 3 diluted 10 times. The blue dots are the PCR partitions resulted positive to amplification of the target; the yellow dots are the PCR partitions negative to amplification of the target.

Figure 2 reports examples of the amplification patterns obtained after cdPCR analysis of some fungal DNA samples.

**Figure 3:**
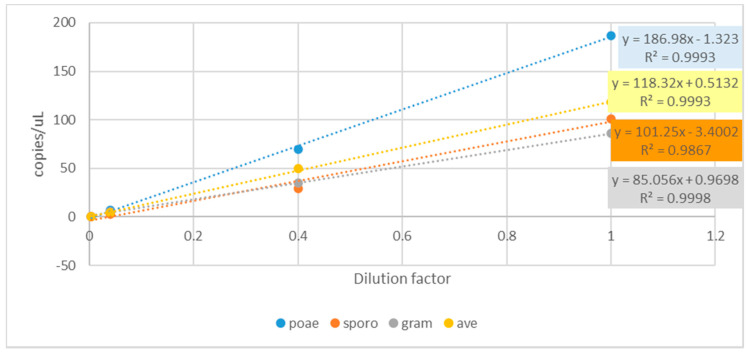
Linear regression among DNA dilution factors vs fungal copies numbers/μL as determined by cdPCR amplification of *F. poae* (blue dots), *F. sporotrichioides* (red dots), *F. graminearum* (grey dots) and *F. avenaceum* (yellow dots).

Linearity between DNA dilution factors and copies/uL (cdPCR determined) has been found for all the assays (Figure 3) as well as high correlation levels between theoretical and cdPCR measured copies/μL for *F. sporotrichioides* (R^2^ = 0.987), *F. graminearum* (R^2^ = 0,999), *F. poae* (R^2^ = 0.999) and *F. avenaceum* (R^2^ = 0.999). 

The Limit of Detection (LOD) and the sensitivity in cdPCR, for the four assays tracking *Fusarium,* were calculated with QuantStudioTM 3D AnalysisSuite dPCR software, as reported in Table 4.

**Figure 4:**
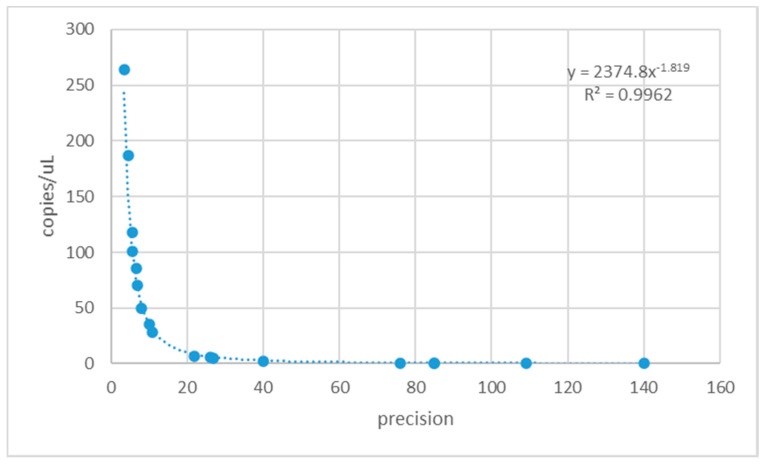
Relationship between precision values and copies/μL determined with the four *Fusarium* detection assays.

Precision refers to the ability of distinguish between two measurements with a certain confidence. The AnalysisSuite TM Software calculates precision as the ratio of the maximum deviation of the confidence interval to the mean value, therefore it expresses the tightness of the confidence interval: the lower the precision, the tighter the confidence interval. In Figure 4, the precisions and the corresponding quantification values obtained amplifying fungal DNA dilutions with the four assays are reported. The highest DNA dilutions move rare target samples below the lower limit of detection and outside the supported dynamic range.

### 3.2. Plant Samples

cdPCR assays were validated on cereal samples naturally infected with mycotoxigenic fungi or spiked with fungal DNA. As previous step to cdPCR analyses, a set of durum wheat and oats samples were analyzed with the qPCR assays described in Table 2 to track fungal species. The rationale behind this preliminary step was to individuate samples naturally contaminated and samples free of fungal contamination and therefore suitable for the preparation of artificially contaminated ones. Three classes were found: i) samples in which no fungal species has been detected; ii) samples contaminated with one *Fusarium* species; iii) samples contaminated with two or more *Fusarium* species. 

Starting from the data, the following two subsets of samples were further analyzed with cdPCR.

- Naturally contaminated samples, belonging to classes ii) and iii);
- Synthetic samples created by spiking plant DNA (extracted from samples found not contaminated) with fungal DNA at different concentrations.

The fungus quantifications in the two subsets of samples obtained with the qPCR and cdPCR assays are reported in Table 5.

The contaminated plant samples belonged to *Triticum durum* and *Avena sativa* species; therefore, a further objective of our study was to develop a duplex assay, able to co-amplify both plant and fungal DNA. The rationale behind the duplex assay’s development is to evaluate the impact of relevant quantity of plant DNA on the functioning of primers and probes of the fungal PCR assays. Moreover, the ratio between the quantity of *Fusarium* and plant DNA can be informative about the infection level in a sample. Two genic targets for durum wheat and oat were selected from the literature or newly developed. The Grano CO2 assay, designed on *TaHd1* gene sequence and developed by Morcia et al. [18] was used to track *Triticum* genus. A new assay (Avena dig assay) was designed on *actin1* gene sequence to track Avena genus. Grano CO2 and Avena dig assays amplification efficiencies, evaluated in qPCR, have values of 99.6 and 111%, respectively. 

The compatibility of the tests to trace the fungal and plant species in cdPCR was evaluated comparing the precision values of the simplex vs. duplex assays. Correlation values ranged from a minimum of 0.97 to 0.99; therefore, the assays are fully compatible and can be organized in duplex mode.

**Figure 5:**
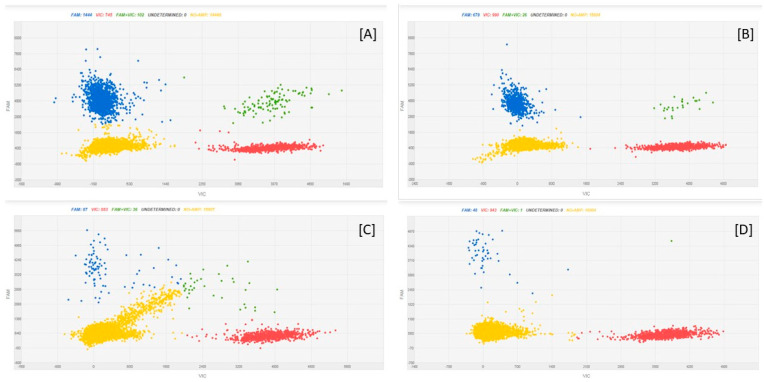
cdPCR amplification plots of oat DNA spiked with *F. avenaceum* DNA dilutions. [**A**] = 20 ng oat DNA + 0.25 ng *F. avenaceum* DNA; [**B**] = 20 ng oat DNA +0.1 ng *F. avenaceum* DNA; [**C**] = 20 ng oat DNA +0.01 ng *F. avenaceum* DNA; [**D**] = 20 ng oat DNA +0.001 ng *F. avenaceum* DNA. The blue dots are the PCR partitions with a positive result, indicating an amplification of the fungal target; the red dots are the partitions which are positive to the plant target; green dots are the partitions in which both the targets were amplified; and the yellow dots are the empty partitions, showing a result that was negative to the amplification of the targets.

Figure 5 reports, as examples, cdPCR plots of oat DNA spiked with *F. avenaceum* DNA dilutions. 

**Figure 6:**
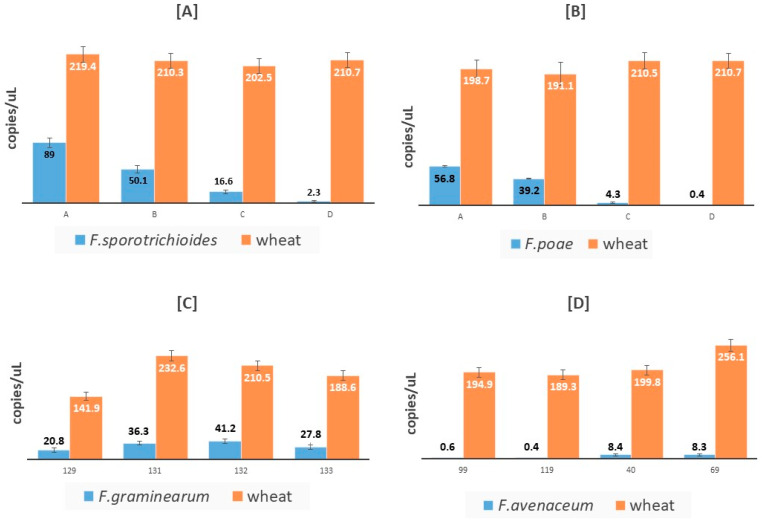
Mean copies/μL of fungal (blue bars) and wheat (red bars) quantified in durum wheat samples spiked with *F. sporotrichioides* [**A**] or with *F. poae* [**B**] and in durum wheat samples found naturally contaminated with *F. graminearum* [**C**] or with *F. avenaceum* [**D**].

Figure 6 reports some examples of the results obtained quantifying with cdPCR the fungal and wheat copies/uL in durum wheat samples spiked with fungal strains (Figure 6A,B) or in naturally contaminated ones (Figure 6C,D). 

## 4. Discussion

In this work we propose four cdPCR assays for detection and quantification of mycotoxigenic *Fusarium*, etiological agents of *Fusarium* Head Blight in small-grain cereals. The assays were organized as duplex assay to simultaneously quantify the fungus and the plant species. The logic behind the development of molecular tools for *Fusarium* diagnosis rely on the possibility to increase fungal control in plants. The fungal DNA can be tracked in the plant during the initial phase of infection, when visible symptoms are absent. Such early diagnosis can mitigate mycotoxin contamination problems in the harvested grains thanks to appropriate fungicidal treatments applied in the right temporal window as well as segregation of highly infected field sectors. We focused on *Fusarium* species worldwide spread in cereal cultivation areas: *F. graminearum* and *F.culmorum*, which are widely recognized as the most important DON producers in small-grain cereals [19], *F. poae* which shows a NIV chemotype although not all isolates produced NIV in vivo [20], *F. sporotrichioides*, a T-2 and HT-2 toxins producer which is frequently isolated in some temperate regions of Europe [21] and *F. avenaceum*, an enniatin and beauverin producer [22].

As already stated, several molecular assays have been recently developed for *Fusarium* diagnosis but, to the best of our knowledge, none based on a digital PCR has been proposed until now. 

Our assays fill this gap, giving the chance to identify and quantify the presence of mycotoxigenic *Fusarium* in small-grain cereal samples with digital PCR technology. Such new assays can be now practically used in *Fusarium* diagnosis. 

As reported by other authors [23,24], dPCR has several advantages in a comparison with qPCR.

➢ As the most advantageous feature, dPCR relies on absolute quantification of the target operated by dPCR; on the contrary, “*results generated from qPCR were relative to calibration curve and were not the actual number of copies in a sample itself*” [23].
➢ Secondarily, the high sample partitioning ensures accurate results even at very low target copy numbers as well as detection of rare targets even in a high background of non-target DNA [24],
➢ Lastly, dPCR is less sensitive to contaminants eventually present in the samples; complex biomolecules such as humic acid can, in fact, significantly inhibit qPCR reactions, but dPCR can overcome this lack thanks to its endpoint quantification [23].

Our cdPCR assays have a LOD ranging from 2 to 13 copies/μL; this level of sensitivity is suitable to *Fusarium* diagnosis purposes in field, for FHB control, for fungicide treatments optimization and breeding purposes. The main disadvantages we encountered, compared with qPCR, are related to the expenses of the analysis, amount of sample analyzed in a certain time. Controversial are the opinions on the commercial cost of dPCR vs qPCR assays. The processivity can be improved by multiplexing, as suggested by Demeke and Dobnik [24]; it is also related to the different instruments available on the market.

Our specific experience highlighted the necessity of specific laboratory skills for both qPCR and cdPCR as well as similar supporting instruments in the laboratory.

In conclusion, our position on the topic is that dPCR has the potential to replace qPCR in some diagnostic fields, e.g., for Genetically Modified Organisms detection [24]. With regard to microbiological routine diagnostics, the two technologies can be considered complementary, and therefore advantageously used in combination. qPCR technology, as previously stated, requires a reference standard curve for quantification, although standardized reference materials of plant pathogens are generally unavailable. Digital PCR, on the contrary, gives an absolute quantification of the molecular target as output and can, therefore, be proposed for characterization of the calibrators needed for standard curves in qPCR analyses. As suggested by other authors [25], dPCR can hypothetically be exploited for the production of calibrators. 

## Figures and Tables

**Table 1:** Primer and probe sequences used in the study to target different *Fusarium* and plant species.

Assay Code	Probe and Primers	Biological Target	Target Gene	Reference
*F. spo*	Pr: FAM-CTGCATCACAACCC-MGB F: GCAAGTCGACCACTGTGAGTACA R: TGAAACTACCCCGCCAAGTC	*F. sporotrichioides*	*tef1* GenBank: MN120771.1	This work
*F. gram/culm*	Pr: FAM-ATCAGTGCTTAAATGCA-MGB F: CAGTAGAGTCGACAAGATCTGCAATC R: TGAAAGTCGCGTAGCTGGAA	*F. graminearum* *F. culmorum*	*Tri* GenBank: MH514957.1	This work
*F. poae*	Pr: FAM-AAAGCGGTCGAGTCTG-MGB F: GCGGCCGCTTTTGTCA R: GCCTTTCCAGCAAGAGATGGT	*F. poae*	*esyn1*	[17]
*F. avetric*	Pr: FAM- CCGTCGAGTCCTCT -MGB F: AGCAGTCGAGTTCGTCAACAGA R: GGCYTTTCCTGCGAACTTG	*F. avenaceum, F.tricinctum*	*esyn1*	[17]
Grano CO2	Pr: VIC- CATGAGCGTGTGCGTG -MGB F: TGCTAACCGTGTGGCATCAC R: GGTACATAGTGCTGCTGCATCTG	*Triticum* genus	*Triticum TaHd1*	[18]
Avena dig	Pr: VIC- ACAATCTTTGCTTGTTCTT-MGB F: TCGTTGATTTTTGGTTGCTTTG R: AGCCTTTGCAATCCACATCTG	*Avena*	*actin 1,* GenBank: AF234528.1	This work

**Table 2:** Optimized amplification conditions applied in qPCR and dPCR assays.

Assay Code	Initial Activation Step	Denaturation/Annealing/Amplification Step	N. of Cycles
*F. sporo*	95 °C, 10 min	95 °C, 30 s; 58 °C, 2 min	47
*F. gram/culm*	95 °C, 10 min	95 °C, 30 s; 60 °C, 2 min	45
*F. poae*	95 °C, 10 min	95 °C, 30 s; 60 °C, 2 min	45
*F. avetric*	95 °C, 10 min	95 °C, 30 s; 59 °C, 2 min	47
Grano CO2	96 °C, 10 min	98 °C, 30 s; 58–60 °C, 2 min	45–47
Avenadig	96 °C, 10 min	95 °C, 30 s; 58–60 °C, 2 min	45–47

**Table 3:** The R2 coefficients and amplification efficiencies of the four assays targeting *Fusarium* species were calculated in qPCR with the standard curve approach, using six calibration points with three PCR replicates each and the formula E = 10^−1^/slope.

qPCR Assay Name	R2 Coefficient	Amplification Efficiency
*F. sporo*	0.984	104%
*F. gram/culm*	0.969	124%
*F. poae*	0.997	100%
*F. avetric*	0.991	106%

**Table 4:** Limit of detection expressed as copies/μL and sensitivity of the four cdPCR assays.

Target	Limit of Detection	Sensitivity
*F. sporotrichioides*	10	0.466%
*F. graminearum*	13	0.6%
*F. poae*	2	0.134%
*F. avenaceum*	8	0.636%

**Table 5:** *Fusarium* quantification values obtained in both naturally contaminated and in spiked samples with qPCR and cdPCR technologies.

Fusarium Strain	Plant Genus	Naturally Contaminated Sample	Spiked Sample	pg of Fungal DNA/50 ng plant DNA (qPCR)	Fungal copies/μL (cdPCR)
*F. sporotrichioides*	*Triticum durum*		X	360 ± 12	89 ± 7.2
*F. sporotrichioides*	*Triticum durum*		X	120 ± 9	50.1 ± 5.2
*F. sporotrichioides*	*Triticum durum*		X	61 ± 3	16.65 ± 3
*F. sporotrichioides*	*Triticum durum*		X	4.1 ± 0.9	2.35 ± 1.1
*F. graminearum/culmorum*	*Triticum durum*	X		34 ± 5	20.8 ± 3.4
*F. graminearum/culmorum*	*Triticum durum*	X		60 ± 2	36.35 ± 4.7
*F. graminearum/culmorum*	*Triticum durum*	X		66 ± 9	41.2 ± 4.9
*F. graminearum/culmorum*	*Triticum durum*	X		57 ± 6	27.8 ± 4.2
*F. poae*	*Triticum durum*	X		1.3 ± 0.2	0.08 ± 0.04
*F. poae*	*Triticum durum*	X		2.1 ± 0.5	0.87 ± 0.1
*F. poae*	*Triticum durum*	X		1.6 ± 0.6	0.91 ± 0.5
*F. poae*	*Triticum durum*		X	126 ± 1.5	56.7 ± 6.4
*F. poae*	*Triticum durum*		X	62 ± 0.9	39.2 ± 7.3
*F. poae*	*Triticum durum*		X	6.2 ± 0.4	4.35 ± 1.6
*F. poae*	*Triticum durum*		X	0.15 ± 0.1	0.40 ± 0.2
*F. avenaceum*	*Triticum durum*	X		1.1 ± 2.1	0.6 ± 0.46
*F. avenaceum*	*Triticum durum*	X		1.6 ± 0.9	0.4 ± 0.3
*F. avenaceum*	*Triticum durum*	X		14 ± 2.3	8.4 ± 2.2
*F. avenaceum*	*Triticum durum*	X		16 ± 1.9	8.3 ± 2.3
*F. avenaceum*	*Triticum durum*		X	300 ± 9.2	118 ± 8.5
*F. avenaceum*	*Triticum durum*		X	120 ± 7.5	50.1 ± 5.4
*F. avenaceum*	*Triticum durum*		X	46 ± 4.6	5.5 ± 1.8
*F. avenaceum*	*Triticum durum*		X	4.8 ± 1.8	0.89 ± 0.71
*F. avenaceum*	*Avena sativa*		X	286 ± 8.2	128 ± 9
*F. avenaceum*	*Avena sativa*		X	111 ± 5.8	55.3 ± 5.8
*F. avenaceum*	*Avena sativa*		X	42 ± 4.2	10.1 ± 2.4
*F. avenaceum*	*Avena sativa*		X	5.9 ± 1.2	3.9 ± 1.5
